# Assessing the availability and quality of COVID-19 mortality data in Europe: a comparative analysis

**DOI:** 10.1093/eurpub/ckad088

**Published:** 2023-06-01

**Authors:** Ivan Marinković, Ana Tramošljanin, Marko Galjak

**Affiliations:** Demographic Research Centre, Institute of Social Sciences, Belgrade, Serbia; Stockholm University Demography Unit, Department of Sociology, Stockholm University, Stockholm, Sweden; Demographic Research Centre, Institute of Social Sciences, Belgrade, Serbia

## Abstract

Researching mortality during the COVID-19 pandemic has been challenging due to methodological inconsistencies and the limited availability of vital statistics data. At the beginning of the pandemic, the World Health Organization recommended daily data publication to inform policy response, but these data were often poor. Final data on COVID-19 deaths in many countries are not yet available, especially for 2021. This report shows that many countries have significant inconsistencies between the preliminary number of deaths and what vital statistics and excess mortality indicate. The inconsistencies in the mortality data raise concerns about the reliability of analyses and public health recommendations.

## Introduction

The COVID-19 pandemic has highlighted issues with tracking mortality data. In the early days of the pandemic, the World Health Organization (WHO) recommended that countries publish daily data on the number of people infected and dead from COVID-19 in order to help member states more effectively and transparently manage the health crisis.^1^ While between-country differences in COVID-19 mortality have been studied,^2^^,^^3^ the differences between preliminary and final civil registration and vital statistics data (CRVS) within countries has not been addressed.

Preliminary data on COVID-19 deaths have been hindered by methodological inconsistencies, variations in definitions, testing policies, making it difficult to compare data across countries.^2^ The availability of CRVS data on mortality by cause of death in 2020 and 2021 was supposed to address these issues, but many countries have been slow to publish this data. As a result, final data on COVID-19 deaths for a significant number of countries are not yet available, especially for the year 2021.

This report provides an overview of available data (as of December 2022) on total deaths and COVID-19 deaths in Europe in 2020 and 2021. We also compare preliminary and final data on COVID-19 deaths. The primary goal of this report is to assess the availability and quality of published mortality data during the pandemic.

## Methods

### Data

We utilize two sources of data: preliminary daily reported COVID-19 deaths aggregated over 2 years and the CRVS data, a well-established source of mortality information. The preliminary data are released daily and can be accessed for the entire year on 1 January of the following year. In contrast, the CRVS pipeline is slower, and many countries have not yet released detailed mortality data for 2020. However, CRVS is the most definitive source of COVID-19 mortality data.^4^ All-cause mortality figures are typically released more quickly and can be used to calculate excess mortality.

Final data on the total number of deaths in 2020 and 2021 are not available in the WHO and Eurostat aggregate databases for many countries. Data on deaths by cause are even less readily available. This report was based on a search of European national statistical offices.

The Supplementary materials contain data for each country along with their sources.

### Indicators

We calculated two measures to assess the correspondence between the preliminary reported COVID-19 mortality data and the final CRVS data, as well as the excess mortality data.

*Inconsistency*: we compare the consistency of preliminary and final data on COVID-19 mortality using relative numbers or indices. The final data serve as the base value (e.g. if they are the same the value will be 100).

*Share*: to assess the quality of the preliminary data, we calculate the share of COVID-19 deaths in 2020 in the total excess mortality for that year. Excess mortality is calculated as the difference between the total deaths in 2020 and the average deaths in 2017–19.

*Data usability*: before the pandemic, the WHO regularly produced mortality data ‘usability’ estimation for countries that report CRVS data, classifying them as having high, medium, low and very low usability scores. In our analysis, we used this classification from the last report (covering years 2000–19)^5^ and the two indicators above to see if countries with low data usability scores tend to have more inconsistent COVID-19 mortality data.

## Results

### Inconsistency of preliminary and final data

**Figure 1:**
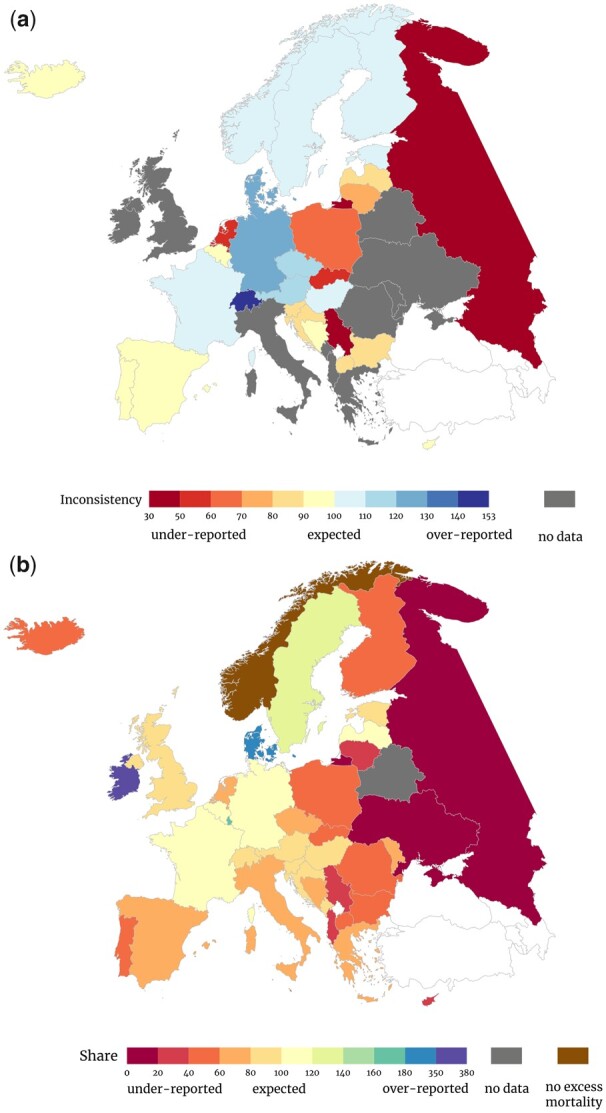
(a) Inconsistency between the preliminary data and the final civil registration and vital statistics COVID-19 mortality data for 2020. (b) Share of preliminary reported COVID-19 deaths in excess mortality for 2020.

In most countries, the preliminary data undercounted the total number of COVID-19 deaths (Figure 1). The data for 2020 show that Serbia and Russia had approximately three times fewer COVID-19 deaths than were later shown in the final data. The Netherlands and Slovakia had about twice as few, Poland had 45% fewer, Lithuania had 26% fewer and other countries usually had under 20% fewer preliminary reported deaths. The data differed very little in Belgium, Sweden, Norway and Iceland. There were also cases of overestimation, with preliminary figures being 20% or higher than the final figures in Switzerland, Germany and Denmark. Data for 2021 are less available, so comparison is possible for fewer countries. Serbia is the only European country where the situation has not changed, with the preliminary data still being three times lower than the final data (Supplementary table S1). In Russia and the Netherlands, the value is also twice as low. The data differ by 20% or less for the rest of the countries. 2021 data are not yet available for as many as 26 out of the 40 selected countries of Europe.

### Share of COVID-19 in excess mortality

The reliability of the preliminary data can be clearly seen when comparing it with excess mortality data. In the first year of the pandemic, preliminary data on COVID-19 mortality explained less than a fifth of the excess deaths in Russia and Ukraine and <30% in Albania and Serbia. In most of Eastern Europe, it explained <50% of excess deaths.

### Data usability

CRVS publication record does not correlate with the inconsistency of preliminary and CRVS data (Supplementary figure S1), as there are countries with CRVS systems with excellent track records that proved to have major inconsistencies. Countries that scored lower on usability before the pandemic tended to undercount the number of COVID-19 deaths in their preliminary data (no such country overcounted).

## Discussion

The quality of preliminary data varies significantly across countries, due to differences in their organizational and control mechanisms. The primary distinction between preliminary and final data lies in their registration time. Additionally, the level of data verification is minimal or absent for preliminary data, while final data undergo a series of checks before publication. The possibility of political intervention in the production or presentation of preliminary data cannot be entirely dismissed. A study indicated that some governments may have underreported preliminary COVID-19 mortality data.^6^ However, further investigation is needed to confirm or refute this possibility.

In certain countries, it was observed that the healthcare systems faced challenges in responding to the pandemic and to simultaneously provide appropriate healthcare services to ‘non-COVID’ patients. While age structure contributes to variations in mortality rates among European countries, there are many other factors at play. Differences in socioeconomic conditions, household composition, access to health care and pandemic response provide possible contextual explanations for these disparities.

Without definitive data on COVID-19 deaths, many analyses and public health recommendations were based on preliminary. Researchers had to use preliminary data from aggregate databases, whose quality is questionable because they can differ significantly from the final data (Figure 1a). Previous research has already flagged some extreme cases as inaccurate, e.g. Serbia^7^ and Russia,^8^ our report indicates that many other countries (e.g. Netherlands, Poland and Denmark) also have yet unexplained inconsistencies. Conducting a comprehensive analysis of data collection processes in each country is necessary for understanding the observed discrepancies and identifying their underlying causes.

The negative impact of using inaccurate data on research, public policy and trust during the pandemic deserves consideration.^9^^,^^10^ Although not all countries have problematic data, many do, and the research community must be mindful of this. It is crucial for the institutions of these countries to improve the accuracy and timeliness of mortality data to enable effective public health responses to future pandemics and health emergencies.

## Supplementary Material

ckad088_Supplementary_Data

## Data Availability

The data underlying this article are available in the article and in its Supplementary material.

## Supplementary data

Key points

- Some countries show significant inconsistencies between their daily reported COVID-19 mortality and what final vital statistics and excess mortality show.
- Many countries across Europe are slow to release their final, vital statistics based, detailed mortality data for 2020 and 2021.
- This report highlights the need to improve the quality and timeliness of mortality data to enable effective public health responses to future pandemics and health emergencies.
