# Supplementary material for: Assessing the availability and quality of COVID-19 mortality data in Europe: a comparative analysis
Source: Eur J Public Health. 2023 Jun 1;33(5):944–6. doi: 10.1093/eurpub/ckad088 (PMC10567233; doi:10.1093/eurpub/ckad088)
Supplement: ckad088_Supplementary_Data [file ckad088_supplementary_data.zip › ckad088_Supplementary_Data/ejph-2023-01-sr-0001-File003.docx]

# Supplementary Information

Methodological notes on the indicators

The way the two indicators we used were calculated are denoted below. The data used to calculate the indicators is given in Table S1. The resulting indicators are provided in Table S2.

$$Inconsistency=\frac{{COVID19 deaths}_{prelim}}{{COVID19 deaths}_{final}}*100$$

$$Share=\frac{{COVID19 deaths}_{prelim}}{{Deaths}_{\mathrm{excess}}}*100$$

Excess deaths used in the Share indicator were calculated based on the pre-pandemic three-year average.

$${Deaths}_{excess}={Deaths}_{expected}-{Deaths}_{observed}$$

Where:

$${Deaths}_{expected}=\frac{{Deaths}_{2017}+{Deaths}_{2018}+{Deaths}_{2019}}{3}$$

Table S1. Countries by the number of deaths, COVID19 deaths, and excess deaths.

| **Country** | **All deaths** | | | **COVID19 deaths** | | | | **Excess deaths** | |
| --- | --- | --- | --- | --- | --- | --- | --- | --- | --- |
|  | **Expected (2017-2019)** | **Observed**  **2020** | **Observed**  **2021** | **Prelim 2020** | **Final 2020** | **Prelim**  **2021** | **Final 2021** | **2020** | **2021** |
| Albania | 21991 | 27605 | 30580 | 1174 |  | 2041 |  | 5614 | 8589 |
| Austria | 83131 | 91599 | 91962 | 7480 | 6491 | 9306 | 7857 | 8468 | 8831 |
| Belarus | 119967 |  |  | 1414 |  | 4147 |  |  |  |
| Belgium | 109243 | 126850 | 112291 | 19749 | 19837 | 8635 | 8563 | 17607 | 3048 |
| Bosnia and Herzegovina | 38184 | 44427 |  | 4050 | 4438 | 9378 |  | 6243 |  |
| Bulgaria | 107511 | 124735 | 148995 | 7515 | 8554 | 23375 | 27588 | 17224 | 41484 |
| Croatia | 52659 | 57023 | 62712 | 3860 | 4478 | 8633 |  | 4364 | 10053 |
| Cyprus | 6001 | 6553 | 7110 | 122 | 131 | 524 |  | 552 | 1109 |
| Czechia | 112238 | 129289 | 139891 | 11888 | 10539 | 24408 |  | 17051 | 27653 |
| Denmark | 54150 | 54645 | 57152 | 1286 | 1070 | 1979 |  | 495 | 3002 |
| Estonia | 15565 | 15811 | 18587 | 229 | 209 | 1703 | 1761 | 246 | 3022 |
| Finland | 54066 | 55488 | 57659 | 592 | 558 | 1136 |  | 1422 | 3593 |
| France | 596522 | 654599 | 639000 | 64004 | 58281 | 56958 | 66282 | 58077 | 42478 |
| Germany | 942222 | 985572 | 1023687 | 50378 | 39758 | 67782 |  | 43350 | 81465 |
| Greece | 123252 | 131084 | 143584 | 4788 |  | 15920 |  | 7832 | 20332 |
| Hungary | 130774 | 141002 | 155621 | 9537 | 8981 | 29649 | 24838 | 10228 | 24847 |
| Iceland | 2250 | 2307 | 2333 | 29 | 30 | 8 |  | 57 | 83 |
| Ireland | 31162 | 31765 | 33055 | 2270 |  | 3787 |  | 603 | 1893 |
| Italy | 633775 | 740317 | 709035 | 73604 |  | 63643 |  | 106542 | 75260 |
| Latvia | 28269 | 28854 | 34600 | 626 | 701 | 3935 |  | 585 | 6331 |
| Lithuania | 38928 | 43547 | 47746 | 1800 | 2266 | 5597 | 7018 | 4619 | 8818 |
| Luxembourg | 4301 | 4609 | 4489 | 502 | 495 | 413 | 417 | 308 | 188 |
| Malta | 3649 | 4084 | 4163 | 219 |  | 258 |  | 435 | 514 |
| Moldova | 36818 | 40717 | 45437 | 3110 |  | 7159 |  | 3899 | 8619 |
| Montenegro | 6541 | 7293 | 9152 | 677 |  | 1724 |  | 752 | 2611 |
| Netherlands | 151912 | 168678 | 170972 | 11296 | 20137 | 9588 | 19562 | 16766 | 19060 |
| North Macedonia | 20170 | 25755 | 28516 | 2488 | 2810 | 5478 |  | 5585 | 8346 |
| Norway | 40765 | 40612 | 42001 | 433 | 414 | 947 | 843 | -153 | 1236 |
| Poland | 408920 | 477355 | 519517 | 28641 | 41442 | 68416 |  | 68435 | 110597 |
| Portugal | 111534 | 123720 | 125185 | 6840 | 7107 | 12094 |  | 12186 | 13651 |
| Romania | 262391 | 298651 | 334910 | 15596 |  | 43118 |  | 36260 | 72519 |
| Russia | 1817781 | 2138586 | 2445509 | 57019 | 163300 | 251841 | 517795 | 320805 | 627728 |
| Serbia | 102278 | 116454 | 136622 | 3163 | 10356 | 9525 | 27742 | 14176 | 34344 |
| Slovakia | 53814 | 59089 | 73461 | 2138 | 3726 | 14497 | 14927 | 5275 | 19647 |
| Slovenia | 20527 | 24016 | 23261 | 2956 | 3390 | 3168 |  | 3489 | 2734 |
| Spain | 423649 | 493776 | 450687 | 54459 | 60358 | 37096 |  | 70127 | 27038 |
| Sweden | 90974 | 98124 | 91958 | 9647 | 9441 | 5716 | 5319 | 7150 | 984 |
| Switzerland | 67280 | 76195 | 71192 | 7528 | 4902 | 4393 |  | 8915 | 3912 |
| Ukraine | 406634 | 616835 | 714263 | 18533 |  | 77366 |  | 210201 | 307629 |
| United Kingdom | 609298 | 689629 | 667042 | 75241 |  | 74682 |  | 80331 | 57744 |

Table S2. Countries by inconsistency of preliminary and final data, share of the preliminary reported deaths in excess mortality and usability of the vital statistics data prior to the pandemic.

| **Country** | **Inconsistency** | | **Share** | | **Data usability (2000-2019)** |
| --- | --- | --- | --- | --- | --- |
|  | **2020** | **2021** | **2020** | **2021** |  |
| Albania |  |  | 20.91 | 23.76 | low |
| Austria | 115.24 | 118.44 | 88.34 | 105.38 | high |
| Belarus |  |  |  |  | high |
| Belgium | 99.56 | 100.84 | 112.17 | 283.33 | high |
| Bosnia and Herzegovina | 91.26 |  | 64.87 |  | low |
| Bulgaria | 87.85 | 84.73 | 43.63 | 56.35 | medium |
| Croatia | 86.20 |  | 88.45 | 85.87 | high |
| Cyprus | 93.13 |  | 22.10 | 47.25 | medium |
| Czechia | 112.80 |  | 69.72 | 88.27 | high |
| Denmark | 120.19 |  | 259.80 | 65.92 | high |
| Estonia | 109.57 | 96.71 | 93.09 | 56.35 | high |
| Finland | 106.09 |  | 41.63 | 31.62 | high |
| France | 109.82 | 85.93 | 110.21 | 134.09 | high |
| Germany | 126.71 |  | 116.21 | 83.20 | high |
| Greece |  |  | 61.13 | 78.30 | medium |
| Hungary | 106.19 | 119.37 | 93.24 | 119.33 | high |
| Iceland | 96.67 |  | 50.88 | 9.64 | high |
| Ireland |  |  | 376.45 | 200.05 | high |
| Italy |  |  | 69.08 | 84.56 | high |
| Latvia | 89.30 |  | 107.01 | 62.15 | high |
| Lithuania | 79.44 | 79.75 | 38.97 | 63.47 | high |
| Luxembourg | 101.41 | 99.04 | 162.99 | 219.68 | high |
| Malta |  |  | 50.34 | 50.19 | high |
| Moldova |  |  | 79.76 | 83.06 | high |
| Montenegro |  |  | 90.03 | 66.03 | low |
| Netherlands | 56.10 | 49.01 | 67.37 | 50.30 | high |
| North Macedonia | 88.54 |  | 44.55 | 65.64 | medium |
| Norway | 104.59 | 112.34 | -283.01 | 76.62 | high |
| Poland | 69.11 |  | 41.85 | 61.86 | medium |
| Portugal | 96.24 |  | 56.13 | 88.59 | high |
| Romania |  |  | 43.01 | 59.46 | high |
| Russia | 34.92 | 48.64 | 17.77 | 40.12 | medium |
| Serbia | 30.54 | 34.33 | 22.31 | 27.73 | high |
| Slovakia | 57.38 | 97.12 | 40.53 | 73.79 | high |
| Slovenia | 87.20 |  | 84.72 | 115.87 | high |
| Spain | 90.23 |  | 77.66 | 137.20 | high |
| Sweden | 102.18 | 107.46 | 134.92 | 580.89 | high |
| Switzerland | 153.57 |  | 84.44 | 112.30 | high |
| Ukraine |  |  | 8.82 | 25.15 | medium |
| United Kingdom |  |  | 93.66 | 129.33 | high |

Inconsistency - of preliminary and final data on COVID-19 mortality using relative numbers, or indices

Share - of COVID-19 deaths in 2020 in the total excess mortality for that year. Excess mortality is calculated as the difference between the total number of deaths in 2020 and the average number of deaths in the period 2017-2019.

Data usability (2000-2019) - refers to the data usability score of vital statists in World Health Organization report *WHO methods and data sources for country-level causes of death 2000-2019* ([https://cdn.who.int/media/docs/default-source/gho-documents/global-health-estimates/ghe2019_cod_methods.pdf Accessed 12 December 2022](https://cdn.who.int/media/docs/default-source/gho-documents/global-health-estimates/ghe2019_cod_methods.pdf%20Accessed%2012%20December%202022))


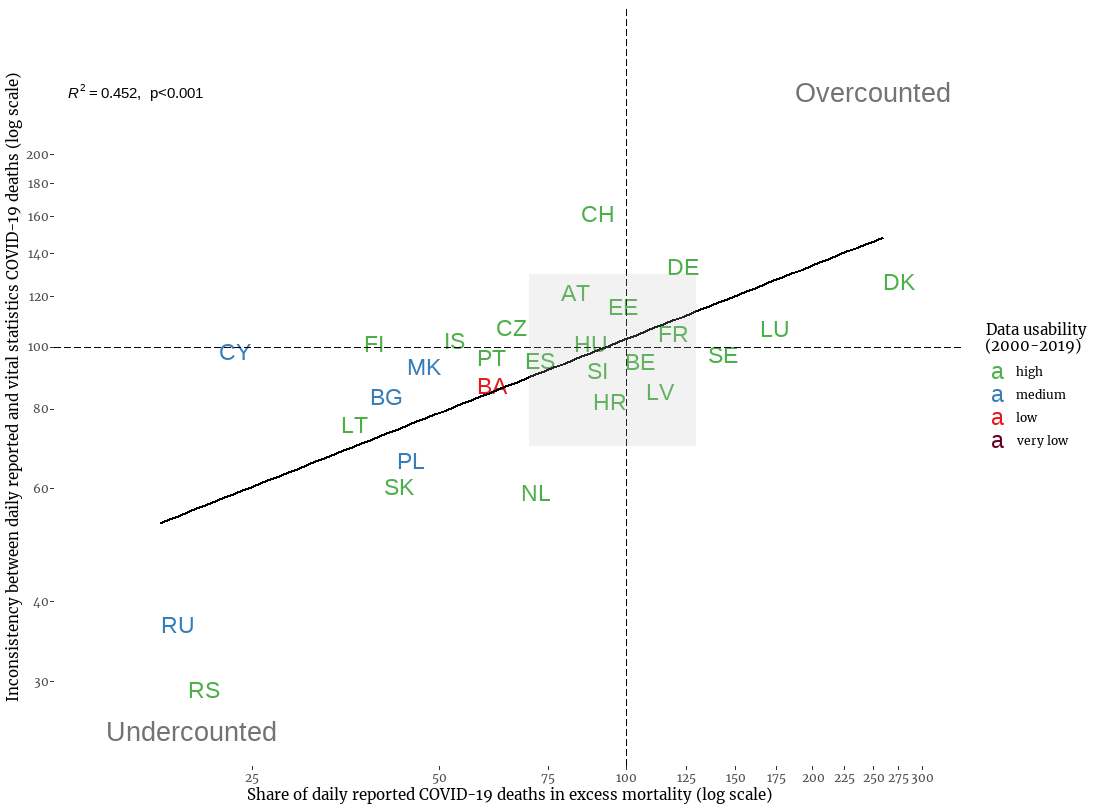


Figure S1. Relationship between the inconsistency between daily reported and vital statistics COVID-19 deaths for and share of daily reported COVID-19 deaths in excess mortality for 2020.

Note: Data usability (2000-2019) - refers to the data usability score of vital statists in World Health Organization report *WHO methods and data sources for country-level causes of death 2000-2019* (https://cdn.who.int/media/docs/default-source/gho-documents/global-health-estimates/ghe2019_cod_methods.pdf Accessed 12 December 2022)

Ideally, all countries in Figure S1 should have indicators of Inconsistency and Share around 100. However, it is expected that there will be deviations, especially for the Share indicator, as not all excess deaths can be directly attributed to COVID-19. The pandemic shock resulted in indirect deaths due to restricted access to adequate healthcare in terms of both quality and timeliness.

The correlation between the two indicators in Figure S1 is influenced by outliers. If we only consider countries within the gray rectangle, whose preliminary, final, and excess mortality data are more or less consistent, we would conclude that there is no such correlation. Contrary to initial expectations, Figure S1 reveals that even countries with a good track record of gathering vital statistics faced challenges in collecting accurate data on COVID-19 deaths during the pandemic. Discrepancies between preliminary, final, and excess mortality data can arise from various reasons, which require individual investigation for each country. Figure S1 should not be used to criticize any specific country but rather as a means to identify potential issues with the collection and reporting of COVID-19 mortality data.
